# Prevalence and factors influencing modern contraceptive use among HIV-positive women in Kilimanjaro region, northern Tanzania

**DOI:** 10.1186/s40834-018-0060-2

**Published:** 2018-05-22

**Authors:** Damian J. Damian, Johnston M. George, Erick Martin, Beatrice Temba, Sia E. Msuya

**Affiliations:** 1 Department of Community Medicine, Kilimanjaro Christian Medical Centre (KCMC), Moshi, Tanzania; 2 Department of Epidemiology & Biostatistics, Institute of Public Health, KCMUCo, P. O. Box 2240, Moshi, Tanzania; 3 Kilimanjaro Christian Medical University College (KCMUCo), Moshi, Tanzania; 4 Department of Community Health, Institute of Public Health, KCMUCo, Moshi, Tanzania

**Keywords:** Modern contraceptives, Contraceptive use, HIV-positive, CTC, Kilimanjaro, Tanzania

## Abstract

**Background:**

Mother-to-Child-Transmission (MTCT) of HIV is still a public health problem in sub-Saharan Africa. The region has a high unmet need for family planning and high unplanned pregnancy rates among HIV-positive women. Most efforts to prevent MTCT of HIV have focused on the third prong, a strategy which offers antiretroviral (ARV) drugs to HIV-infected pregnant women and their exposed infants. However, the effective use of contraceptives to prevent unplanned pregnancies among women living with HIV is more effective in reducing HIV MTCT. This study aimed at determining the prevalence and factors influencing modern contraceptive use among HIV-positive women in northern Tanzania.

**Methods:**

This was a cross-sectional study conducted between January and June 2014 in three selected districts of Kilimanjaro region, Tanzania. Data were collected during face-to-face interviews with HIV-positive women attending Care and Treatment Clinics (CTC) in the selected districts. Multivariate logistic regression analysis was used to determine independent predictors of modern contraceptive use.

**Results:**

In total 672 HIV-positive women were enrolled. Their mean age was 36.4 years (±7.7). Fifty four percent (362) were currently using modern contraceptives, and the most common method used was male condoms 76% (275) followed by Depo-Provera 28% (101). A total of 33% (121) of the users reported dual contraceptive use. Women with primary education [Adjusted Odds Ratio (AOR) = 7.54, 95% Confidence Interval (CI): 1.51–17.48, *P* = 0.014]; post-secondary [AOR = 6.23, 95% CI: 1.14–14.07, *P* = 0.035]; not currently on ARVs [AOR = 11.29, 95% CI: 2.60–19.94, *P* = 0.001]; currently sexually active [AOR = 8.40, 95% CI: 4.47–15.78, *P* < 0.001]; ever discussed contraceptive use with partner [AOR = 3.68, 95% CI: 1.67–8.11, *P* = 0.001]; and being counseled on dual contraceptive use at CTC [AOR = 2.94, 95% CI: 1.66–5.23, *P* < 0.001]; had significantly higher odds of currently using modern contraceptive methods.

**Conclusions:**

Given the population studied, the prevalence of modern contraceptive use was low. Strategies are required to increase the use of dual and long-term contraceptive methods among women who do not want more children in order to reduce MTCT, and to improve maternal and child health in the region. Programme managers and health care providers need to identify counseling strategies that are specific to HIV-positive women that not only impart knowledge on contraceptives, but also address the issue of responsibility for influencing HIV transmission in the community.

## Background

Since the 1960s, family planning (FP) programmes have helped women worldwide to avoid millions of unintended pregnancies often associated with high risk abortions and with maternal, newborn and child morbidity and mortality [1]. Globally, about 220 million women have an unmet need for FP and 80 million unplanned pregnancies occur each year [2, 3]. In sub-Saharan Africa (SSA), around 14 million unintended pregnancies occur yearly [4]. Investing in FP to prevent unwanted pregnancies has the potential of averting 13% of maternal, newborn and child deaths [5].

Recently, the use of FP has been seen to be of high benefit to women living with HIV [1, 6]. As compared to women in the general population, HIV-positive women have high unwanted pregnancy rates (51–90%), especially in SSA region [7]. Prevention of unintended pregnancies among HIV-infected women has a vital role in the prevention of mother-to-child-transmission (MTCT) of HIV. Reducing unintended pregnancies among HIV-positive women through FP reduces the number of HIV-exposed infants born from HIV-positive women and ultimately decreases MTCT [1, 6]. Prevention of unwanted pregnancies also reduces the vulnerability of women and infants to morbidity and mortality related to pregnancies [6, 8]. FP has also proven to be a cost-effective strategy for the prevention of HIV transmission, as contraception costs are less than the cost of drugs used in the Prevention of Mother-To-Child Transmission (PMTCT) [8, 9].

For the elimination of MTCT of HIV, WHO recommends a comprehensive PMTCT strategy with the 4 prongs: 1) Primary prevention of HIV infection among women of childbearing age; 2) FP for preventing unintended pregnancies among HIV-infected women; 3) preventing HIV transmission from HIV-infected women to their infants; and 4) treatment, care and support for HIV-infected women and their children [10]. However, PMTCT of HIV in most SSA countries including Tanzania is based on the third prong which is usually complex, labour and resource intensive [11]. This has led to the second prong gaining recognition as having a vital role in PMTCT.

The mathematical projection from 2009 on the burden of paediatric HIV indicated the synergistic effect of FP on reducing the number of HIV-positive pregnancies. The model showed that while HIV services to prevent mother-to-child transmission averted an estimated 8.1% of vertical infections, FP had the potential to avert 19.7% [7]. According to the model, unintended pregnancies accounted for 21.3% of new paediatric infections [6]. If all women wanting to avoid pregnancy used modern contraceptive methods, unintended pregnancies would decline by 71% [12]. There is therefore the need to improve strategies to raise FP use among HIV-positive women.

Despite the benefits, the unmet need for FP in most SSA countries is high both in general population [13–16] and among HIV-positive women [16–21]. In Tanzania, the unmet need for FP has remained unchanged over the past two decades (22–24%) [14]. In 2015–16, the Contraceptive Prevalence Rate (CPR) among currently married women (15–49 years) was 32%, while the national target by the end of 2015 was 60% [14]. The consequence of low CPR and a high unmet need for FP in this context is a high number of unwanted/unplanned pregnancies. High unwanted pregnancy rates among people living with HIV can further be linked with risk of exposing children to HIV infection unnecessarily [10].

While there is data on trends and current levels of CPR, unmet need, fertility rates and HIV prevalence from various surveys in Tanzania; there is limited information on FP use in HIV-positive women. Understanding the prevalence, patterns and factors influencing modern contraceptive use among HIV-positive women of reproductive age is critical to the expansion of comprehensive HIV prevention programmes, targeted at achieving a reduction in unwanted pregnancies and decreasing the incidence of HIV-infected children.

## Methods

### Study design and site

This was a facility-based cross-sectional study conducted between January and June 2014 among HIV-positive women aged 15–49 in three districts of the Kilimanjaro region, northern Tanzania. The Kilimanjaro region is located in the northern zone of the country and is the home of Africa’s highest mountain, Mt. Kilimanjaro. The region has a population of 1,640,087 people, which is 3.6% of Tanzania’s population [22]. The region has seven districts from which Hai, Moshi urban and Mwanga districts were selected for this study. During the study period, the national HIV prevalence was estimated to be 5.1% [23]. Prior to this study, the regional HIV prevalence was estimated to be 3.8%; with Hai district 5.6%, Mwanga 4.2% and Moshi urban 3.6% respectively [23].

### Study population and enrolment procedures

Three districts with a high HIV prevalence in the region were selected, i.e. Hai, Mwanga and Moshi urban. A simple random sampling technique was used to select health facilities with Care and Treatment Clinics (CTCs) from the three districts. Eight, six and five facilities were selected from Moshi urban, Hai and Mwanga districts respectively. All HIV-positive women aged 15–49 attending a CTC who were eligible to participate and those meeting the inclusion criteria were invited to participate. We excluded HIV-positive women aged 15–49 who had a hysterectomy or did not consent to participate in the study. After obtaining participants’ informed consent, a trained research assistant administered a questionnaire during face-to-face interviews. The collected information included: socio-demographic information (age, education level, marital status, occupation); individual health (time since diagnosed with HIV, current CD4 count, whether was on ART drugs); sexual and reproductive health history (parity, sexual activity, condom use); and methods and pattern of FP use (ever heard of FP, ever used FP, currently using FP). Information on facility factors, partner communication and FP challenges was also collected.

### Variables and outcome measures

In this study, modern contraceptive use was the main study outcome and was defined as using any of the following methods to delay pregnancy i.e. female sterilization, intra-uterine device, injectables (depo-provera), implants, pills, condoms and emergency contraception.

### Statistical analysis

Data were entered, cleaned and analysed using Statistical Package for Social Science (SPSS) version 23.0 (SPSS, Chicago, IL, USA). Descriptive statistics were used to summarise data. Bivariate logistic regression analysis was used to examine the association between current modern contraceptive use and predictors of interest. Multivariate logistic regression analysis was used to determine independent predictors of current modern contraceptive use. *P*-value was considered statistically significant at 5% level. In a multivariate logistic regression analyses, the results were adjusted for marital status, income, intention to have more children, ever discussed with partner on the number of children, ever received counseling on modern contraceptive in CTC and disclosure of HIV status to partner.

## Results

### General characteristics of the study participants

#### Socio-demographic and economic characteristics of the participants

Out of 680 HIV-positive women approached to participate in this study, 672 gave consent, giving a response rate of 98.8%. The mean (±standard deviation) age of participants at enrolment was 36.4 (±7.7) years. More than half of the study participants were aged between 35 and 49 years (61.8%), not currently married (54.6%), had primary education (76.9%), were in informal employment (88%) and living together with partner (87.3%). Table 1 shows the socio-demographic and economic characteristics of participants.

**Table 1:** Socio-demographic and economic characteristics of participants (*N* = 672)

Characteristic	*n* (%)
Age category (years):	
15–24	56 (8.3)
25–34	201 (29.9)
35+	415 (61.8)
Current marital status:	
Currently in union	305 (45.4)
Never in union	127 (18.9)
Formerly in union	240 (35.7)
Education level:	
No formal education	22 (3.3)
Primary education	517 (76.9)
Secondary or higher education	133 (19.8)
Average daily income (*n* = 511):	
≤ $1 per day	192 (37.6)
> $1 per day	319 (62.4)
Employed and receive regular salary:	
Yes	82 (12.2)
No	590 (87.8)
District:	
Moshi urban	305 (45.4)
Hai	187 (27.8)
Mwanga	180 (26.8)
Living arrangements:	
Living together with the partner	270 (87.4)
Visits	35 (11.3)
Living apart for more than 6 months	4 (1.3)

#### Clinical, sexual and reproductive health, couple communications and counseling

Almost half of study participants (49.0%) were diagnosed with HIV between 1 and 4 years prior to enrolment in the study. The majority of participants were on ARVs (89.1%), sexually active (60.4%), and 76.8% had multiple lifetime sexual partners. The vast majority of women (90.3%) reported to have had a child and nearly one quarter (23.8%) were intending to have more children in future. Less than half of the study participants (45.5%) reported to have ever discussed modern contraceptive use with their partners and the number of children they both wanted (42.3%). More than half (55.5%) of the study participants reported having ever received counseling on modern contraceptive use at their respective CTCs, and among those counseled, 64.9% were counseled on dual contraceptive use. Table 2 depicts clinical, sexual and reproductive health, and couple communications characteristics of participants.

**Table 2:** Clinical, sexual and reproductive health, couple communication and counseling characteristics (*N* = 672)

Characteristics	*n* (%)
Duration since diagnosed with HIV:	
< 1 year	35 (5.3)
1–4 years	325 (49.0)
5 years or more	303 (45.7)
Currently on ARVs:	
Yes	599 (89.1)
No	73 (10.9)
Duration on ARVs:	
< 1 year	166 (27.7)
1–4 years	210 (35.1)
5 years or more	223 (37.2)
Currently sexually active:	
No	266 (39.6)
Yes	406 (60.4)
Number of lifetime sexual partner(s):	
Single	156 (23.2)
Multiple	516 (76.8)
Number of sexual partner(s) in past 12 months:	
Single	625 (93.0)
Multiple	47 (7.0)
Have children:	
No	65 (9.7)
Yes	607 (90.3)
Intend to have more children:	
No	512 (76.2)
Yes	160 (23.8)
Ever discussed with partner on number of children:	
No	388 (57.7)
Yes	284 (42.3)
Ever discussed with partner on modern contraceptive use:	
No	366 (54.5)
Yes	306 (45.5)
Ever received counseling on modern contraceptives at CTC:	
No	299 (44.5)
Yes	373 (55.5)
Ever counseled on dual contraceptives at CTC:	
No	131 (35.1)
Yes	242 (64.9)
Ever given modern contraceptives at CTC:	
No	368 (54.8)
Yes	304 (45.2)

### Prevalence of modern contraceptive use

The majority of participants (84%) reported that they had ever used modern contraceptive methods. The prevalence of current modern contraceptive use was 53.9% (*n* = 362). Of the 362 users, 121 (33.4%) reported using dual contraceptives (i.e. condoms and any modern contraceptive method). Of those who reported current modern contraceptive use, more than one quarter 98 (27.1%) reported that their partners were not aware of it.

### Types of modern contraceptive methods used

**Fig. 1:**
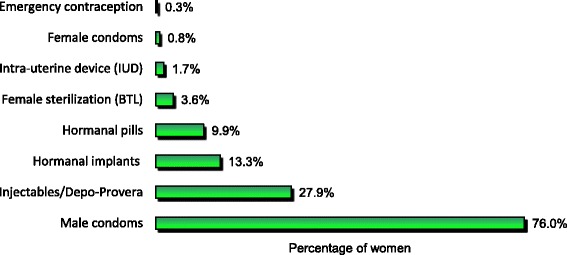
Types of modern contraceptive methods used by study participants (*n* = 362)

The most common used modern contraceptives methods among the 362 women were male condoms (76.0%), Depo-Provera (28.2%) and implants (13.3%). Figure 1 shows types of modern contraceptive methods used by the study participants.

### Factors influencing current modern contraceptive use

**Fig. 2:**
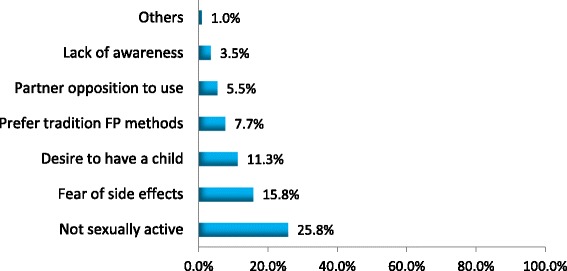
Common reasons for not using modern contraceptive methods (*n* = 310)

Table 3 shows the results of logistic regression analysis of the independent predictors of current modern contraceptive use. In a multivariate analysis woman with primary education had a 8-fold increase in odds of current modern contraceptive use compared to those who never attended school (AOR = 7.54, 95% CI: 1.51–17.48), *P* = 0.014). Those with secondary or higher education, the odds of current modern contraceptive use was 6 times higher than those who never attended school (AOR = 6.23, 95% CI: 1.14–14.07), *P* = 0.035). Not currently on ARVs (AOR = 11.29, 95% CI: 2.60–19.94, *P* = 0.001); currently sexually active (AOR = 8.40, 95% CI: 4.47–15.78, *P* < 0.001); discussed modern contraceptive use with a partner (AOR = 3.68, 95% CI: 1.67–8.11, P = 0.001) and being counseled on dual contraceptive methods at a CTC (AOR = 2.94, 95% CI: 1.66–5.23, *P* < 0.001) had significantly higher odds of current modern contraceptive use. For brevity, the common reasons for modern contraceptive non-use among study participants are presented in Fig. 2.

**Table 3:** Factors influencing current modern contraceptive use (*N* = 672)

Characteristics	Modern contraceptive use (%)	COR¥ (95% CI)	AOR^a^ (95% CI)
*Education level*			
Never attended to school	31.8	1	1
Primary education	52.6	2.38 (0.95–5.93)	23.83 (2.84–331.45)^*^
Secondary/higher education	62.4	3.56 (1.36–9.32)	13.35 (1.50–187.80)^*^
*Marital status*			
Never in union	53.5	1	
Currently in union	71.8	2.21 (1.44–3.39)^*^	
Formerly union	31.3	0.39 (0.25–0.61)^*^	
*Level of daily income (n = 511)*			
≤ $1	49.5	1	
> $1	58.6	1.45 (1.01–2.07)^*^	
*Current on ARV*			
Yes	52.3	1	1
No	67.1	1.87 (1.12–3.12)^*^	19.21 (2.60–141.75)^**^
*Intend to have more children*			
No	59.4	1	
Yes	68.1	2.18 (1.50–3.18)^*^	
*Currently sexually active*			
No	19.2	1	1
Yes	76.6	13.80 (9.42–20.22)^**^	12.29 (5.62–26.89)^**^
*Ever discussed with partner on modern contraceptive use*			
No	39.1	1	1
Yes	71.6	3.93 (2.84–5.43) ^**^	5.26 (1.94–14.26)^*^
*Ever discussed with partner on number of children*			
No	45.6	1	
Yes	65.1	2.23 (1.63–3.05) ^**^	
*Received counseling on contraceptives at CTC*			
No	36.8	1	
Yes	67.6	3.58 (2.60–4.93) ^**^	
*Counseled on dual contraceptives at CTC (n = 373)*			
No	50.4	1	1
Yes	76.9	3.27 (2.08–5.15) ^**^	2.72 (1.29–5.76)^**^
*Disclosed HIV status to partner*			
No	40.8	1	
Yes	62.8	2.44 (1.78–3.35) ^**^	

*¥COR* Crude odds ratio, *AOR* Adjusted Odds Ratio

*Indicates *p*-value < 0.05, **Indicates *p*-value < 0.001

^a^The results are adjusted for marital status, income, intention to have more children, ever discussed with partner on the number of children, ever received counseling on modern contraceptive in CTC and disclosure of HIV status to partner

## Discussion

The results of this study showed that slightly more than half (54%) of the HIV-positive women between the ages of 15 and 49 were currently using modern contraceptive methods. The prevalence of current modern contraceptive use among HIV-positive women in Kilimanjaro was higher than the national (32%) and the regional (47.8% - Kilimanjaro region) prevalence in the general population [14]. It is also higher than that observed among HIV-positive women in Ethiopia (46%) [24], Rwanda (43%) [25], Ghana (43%) [26], Lesotho (35%) [17] and Uganda (28%) [27] but lower than that in women on ART in Zambia (69%) [18] and among women attending CTC care in Dar es Salaam (68.5%) [28]. A higher contraceptive use among HIV-positive women than in the general population may be due to comprehensive counseling and guidance these women receive in various HIV interventions e.g. PMTCT and CTC programmes. A facility-based study conducted in Thailand showed that the vast majority (92%) of women on a PMTCT programme were currently using modern contraceptive methods. This was higher than among those who were referred to HIV CTCs [29]. In Tanzania, most of the women attending a CTC have been referred through PMTCT programmes [30]. Through this programme (PMTCT), counseling on prevention of vertical and horizontal HIV transmission is strongly emphasised. In addition, the majority of women in this study (89%) were currently on ARVs. This group receives intensive counseling on ARV adherence and pregnancy preventions. This might have influenced the high prevalence (as compared to the general population) of current modern contraceptive use in this study.

The use of dual contraceptive methods included correct and consistent use of condoms in conjunction with another type of modern contraceptive method. Evidence from literature has shown its effectiveness (dual methods) in preventing both horizontal and vertical HIV transmission, STIs and pregnancy [5, 6, 8]. The prevalence of dual contraceptive use in this study (33%) is an increase from 19% reported by Antelman et al. among women living with HIV attending HIV clinical care in Dar es Salaam Tanzania in 2001 [19]. The prevalence of dual contraceptive use among women who reported using modern contraceptive methods was relatively higher compared to data from USA (7%) [31], Cambodia (17%) [32], Zambia (25%) [18] and Uganda (4%) [33]; but lower than that reported in Ethiopia (60%) [24] and similar to the recent reports in Dar Es Salaam, Tanzania (32.7%) [28]. The finding that only a third of those using modern contraceptives are using dual methods was in the background that most women (65%) reported to have received counseling on dual contraceptive methods in a CTC. It can be argued that counseling alone is probably not sufficient to improve dual contraceptive use, other novel interventions needs to be developed and tested.

This study showed male condoms to be the most common method of modern contraceptive used (76%) followed by Depo-Provera (28%) and implants (13%). Despite condoms being effective in preventing HIV transmission, the use of alternative effective contraceptive methods to prevent pregnancy was low in this setting. These findings are consistent with data obtained in Ghana, Nigeria, Zambia, Rwanda, Kenya and Dar Es Salaam, Tanzania where male condoms were the most common method of contraceptive used (80%, 53%, 60%, 30%, 20% and 43% respectively) [18, 26, 28, 34, 35]. However, these results are contrary to the results from the Tanzania Demographic and Health Survey whereby in Kilimanjaro region, injectables (21%) followed by implants (10%) were the most common contraceptive methods used by currently married women aged 15–49 [36]. Other results contrary to our findings were observed from Malawi whereas injectables (20%) followed by condoms (13%) [20] were the most common methods of contraceptive used, and in Ethiopia where injectables (71%) were followed by male condoms (48%) [37]. It seems possible that the male condom is a method of contraceptive used frequently by most study participants, as they are more easily available and accessible in the society than other long-term contraceptive methods. In this setting, condoms are obtainable in drug dispensing units, clinics, supermarkets and other marketing areas. Also, the fact that 16% of women who did not use certain modern contraceptive methods due to their fear of side effects, might have favoured the use of the male condom.

Couple communication was a strong predictor of modern contraceptive use in this study. HIV-positive women who reported of ever discussing with the partner on any of the Sexual and Reproductive Health (SRH) issues such as: number of children they wanted, contraceptive use or HIV testing, had significantly higher odds of using modern contraceptives than others. There is a need to introduce couple-communication interventions and interventions that increase the skills of women in negotiating and communicating with partners on SRH issues. Couple communication has not only shown to increase modern contraceptive use in India, Zambia and Malawi [38, 39], but it has also known to improve other maternal and child health indicators like adherence to ART and PMTCT interventions, minimizing loss to follow up in ART/PMTCT programs as well as improving HIV disclosure [40].

Report of receiving counseling on contraceptives and on dual methods, as well as availability of contraceptive methods at CTC were the health facility factors associated with modern contraceptive use in this study. These findings were consistent with report by Polisi et al. in the Western Ethiopia in 2014 [21]. Tanzania developed guidelines on integration of FP services into HIV CTC in 2013. Adherence to this recommendation is not known, but evidence of this work highlight the need to have a ‘supermarket’ approach in that all the centres offering HIV care and treatment services should also offer FP services in the same setting to minimise referrals. Integration of FP services and counseling in CTC improve contraceptive uptakes as evidenced by both experimental and observational studies in different settings [41–44].

The generalisability of these results is subject to certain limitations. For instance, contraceptive use was based on self-report by the women i.e. no method was available to confirm the reported information. However, women might have reported what they thought research assistants wanted to hear, or what was perceived as socially correct, which would have underestimated or overestimated the prevalence obtained in this study. The major strength of this study lies in the population studied which represent both rural and urban settings. The information can fairly represent HIV-positive women in Kilimanjaro region. Another key strength is the high response rate by the study participants.

## Conclusion

Almost one in two women in this study who knew their HIV status were not using any barrier or hormonal methods to prevent unwanted pregnancies or HIV transmission to uninfected partners. The consequence of low CPR in this context and a high unmet need for FP is a high number of unwanted/unplanned pregnancies which can further be linked with risk of exposing children to HIV infection unnecessarily. Programme managers and health care providers need to identify counseling strategies that are specific to HIV-positive women that not only impart knowledge on contraceptives, but also address the issue of responsibility for influencing HIV transmission in the community. Planners need to come up with strategies involving couple communications so as to increase the use of adequate and effective contraceptives methods in preventing unwanted pregnancies in this population.

## Abbreviations

AOR: Adjusted odds ratio
CI: Confidence interval
COR: Crude odds ratio
CPR: Contraceptive prevalence rate
CTC: Care and treatment clinics
FP: Family planning
MTCT: Mother-to-child-transmission
PMTCT: Prevention of mother-to-child transmission
SSA: Sub-Saharan Africa
